# An In-Reflection Strain Sensing Head Based on a Hi-Bi Photonic Crystal Fiber

**DOI:** 10.3390/s130708095

**Published:** 2013-06-25

**Authors:** Sergio Rota-Rodrigo, Ana M. R. Pinto, Mikel Bravo, Manuel Lopez-Amo

**Affiliations:** Department of Electric and Electronic Engineering, Universidad Pública de Navarra, Pamplona 31006, Spain; E-Mails: anamargarida.rodrigues@unavarra.es (A.M.R.P.); mikel.bravo@unavarra.es (M.B.); mla@unavarra.es (M.L.-A.)

**Keywords:** optical fiber sensor, photonic crystal fiber, strain sensor, interferometry

## Abstract

A photonic crystal fiber-based sensing head is proposed for strain measurements. The sensor comprises a Hi-Bi PCF sensing head to measure interferometric signals in-reflection. An experimental background study of the sensing head is conducted through an optical backscatter reflectometer confirming the theoretical predictions, also included. A cost effective setup is proposed where a laser is used as illumination source, which allows accurate high precision strain measurements. Thus, a sensitivity of ∼7.96 dB/ms was achieved in a linear region of 1,200 με.

## 1. Introduction

There are a number of applications of practical interest in which the monitoring of strain-induced changes is important, such as experimental mechanics, aeronautics, metallurgy and health monitoring of complex structures, among others. These applications need continuous monitoring, aiming to control and prevent accidents or abnormal states early in time. Through the monitoring of structures, maintenance and rehabilitation advice can be provided, opening the possibility to avoid casualties [1]. In order to meet the increasing measurement requirements of modern industry, different types of strain sensors based on fiber-optic techniques have been developed. Fiber-optics have a number of characteristics that make them very appealing for sensing purposes, such as immunity to electromagnetic interference, light weight, remote sensing ability, multiplexing capability, and the ability for continuous *in situ* measurement [2]. Photonic crystal fibers (PCFs) are a recently developed class of optical fibers [3], which present a geometry characterized by a periodic arrangement of air-holes running along the entire length of the fiber, centered on a solid or hollow-core. The major difference between PCFs and single mode fibers (SMFs) relies on the fact that the waveguide properties of photonic crystal fibers are not due to spatially varying glass composition, as in conventional fibers, but from an arrangement of very tiny and closely spaced air-holes which go through the whole fiber length. In contrast with standard optical fibers, photonic crystal fibers can be made of a single material and have several geometric parameters that can be manipulated offering great flexibility of design. As such, PCFs present a diversity of new and improved features when compared to common SMFs, introducing innovative solutions in the sensing field [4].

Several strain sensors based on PCFs have been developed. Some modal interferometers have been accomplished using PCFs to measure strain or displacement: by tapering solid-core silica PCFs [5]; or by constructing a sensing head with a sensitivity of ∼2.8 pm/με through splicing a piece of PCF to a SMF and interrogating it with a LED and a miniature spectrometer [6]; or even throughout a core offset at one of the joints of a SMF-PCF-SMF structure with 0.0024 dB/μm of sensitivity [7]. Other authors reported strain sensors that used highly birefringent (Hi-Bi) PCF based Sagnac interferometers showing temperature insensitivity, using a wavelength based measurement (∼1.11 pm/με) [8] and a power based measurement (∼2.7 dB/mε to 3.2 dB/mε of sensitivity) [9]. Displacement sensors were also reported using a Hi-Bi PCF in a Sagnac interferometer with a sensitivity of 0.283 nm/mm [10], and using a three-hole suspended-core fiber in a high precision Sagnac configuration (∼0.45 μm) [11]. In addition, a strain sensor based in a Mach-Zehnder interferometer was accomplished by splicing a short length of PCF between two SMFs with collapsed air holes over a short region in the two splicing points (sensitivity of ∼0.21 μs^−1^/mε) [12]. A miniature in-line Fabry-Perot interferometer was as well obtained for strain sensing by splicing a small length of hollow-core photonic bandgap fiber between two SMFs in order to obtain a strain sensitivity of 1.55 pm/με [13]. Furthermore, a strain sensor was obtained based in a birefringent interferometer fabricated by an all-silica Hi-Bi PCF in transmission with a sensitivity of 1.3 pm/με [14].

In this work, an in-reflection interferometric Hi-Bi PCF sensing head for strain measurement is proposed. A study of the sensing head characteristics is shown, where a theoretical study is in accordance with the experimental data obtained through a high resolution optical backscatter reflectometer. Strain sensing is carried out using an accessible setup, where the interference signal is obtained through an in-line fiber polarizer.

## 2. Operation Principle

The operation principle of the proposed sensing system is based on two main properties of the Hi-Bi PCF: high birefringence and low temperature sensitivity. When light is launched into a highly birefringent fiber the difference in velocities between the two birefringent axes causes the resultant polarization state to vary along the length of the fiber in a controlled manner. The beat length (*L_B_*) is a measure of the birefringence, or ability to preserve polarization. The beat length is defined as the distance over which the polarization rotates through 360 degrees:

(1) $$L_{B}=\frac{\lambda }{b}$$

where λ is the wavelength at which the beat length is measured and *b* is the birefringence of the fiber. Since the sensing head works in reflection, the interferometric signal is proportional to twice the fiber length and its wavelength dependence can be expressed by Equation (2), where *l* is the fiber length, *A* is the amplitude and *ϕ* is the total phase:

(2) $$R{\left(\lambda \right)}_{dB}=10\mathrm{Log}{\left[A\cdot \mathrm{Cos}\left(2\pi \cdot \frac{2l\cdot b}{\lambda }-\phi \right)\right]}^{2}$$

The total phase is defined as *ϕ* = *ϕ*_0_+*Δϕ*. Where *ϕ_0_* is the initial phase and *Δϕ* is the phase change induced by external perturbations. When strain is applied to the fiber, the phase variation will be given by:

(3) $$\Delta \phi (\lambda )=\frac{2\pi }{\lambda }\cdot (\Delta b\cdot 2l+2\Delta l\cdot b)$$

where *Δl* and *Δb* are the length and birefringence variations, respectively.

The low temperature sensitivity characteristic is a direct consequence of single material fabrication. Conventional optical fibers contain two different materials with different thermal (thermal expansion coefficient) and mechanical properties (Young's modulus and Poisson's ratio), which will generate high thermal stress when the fiber is subjected to temperature variations, consequently changing the birefringence of the fundamental mode. Since the Hi-Bi PCF is made of a single material, it will not present thermal stress, and thus, it is not surprising that Hi-Bi PCF temperature associated variations were experimentally measured to be negligible [15].

## 3. Sensing Head Characterization

**Figure 1.**
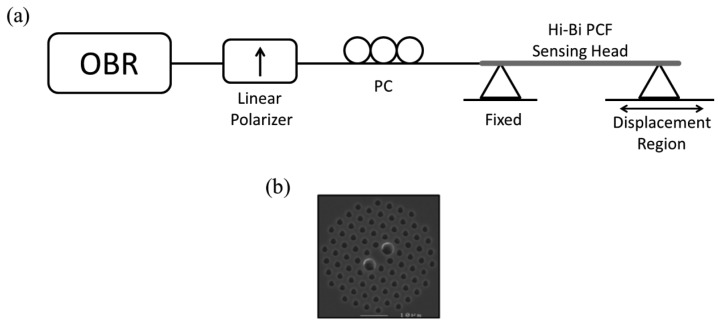
(**a**) Schematic of the experimental setup used to characterize the Hi-Bi PCF sensing head and (**b**) optical microscopic picture of the Hi-Bi PCF cross-section.

The Hi-Bi PCF sensing head was obtained by splicing one end of ∼20.8 cm Hi-Bi PCF to a SMF (maximum loss of 2 dB) and cleaving the other end. The Hi-Bi PCF is a polarization maintaining photonic crystal fiber (PM-1550-01 from NKT Photonics, Birkerød, Denmark) with a beat length of ∼3.65 mm at 1,550 nm and an attenuation of 1.0 dB/Km (a cross section photograph can be seen in the inset of Figure 1). Figure 1 presents the characterization setup using an optical backscattering reflectometer (OBR), a linear polarizer, a polarization controller (PC) and the Hi-Bi PCF sensing head. The OBR used was developed by Luna Technologies and presents characteristics such as high spatial resolution (up to ∼10 μm) for different measurable magnitudes, such as amplitude, polarization states and return loss in time and frequency domains.

In the experimental setup depicted in Figure 1, the linear polarizer converts the polarization state of the source light into a linear one, while the polarization controller allows one to adjust the alignment angle with the PCF. When the light propagates along the PCF, a phase shift is generated between the two birefringent axes due to its own birefringence.

**Figure 2.**
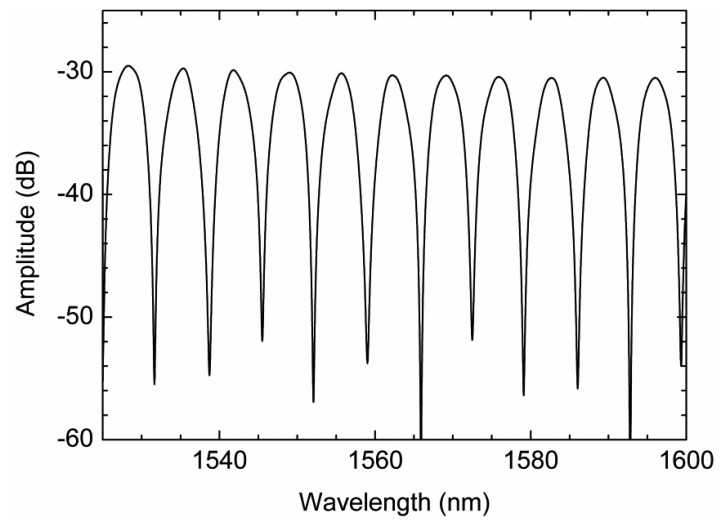
Reflected spectrum of the Hi-Bi PCF interferometer in a relaxed position, when no external force is induced on the sensing head.

The reflected light passes again through the linear polarizer producing the interference between the retarded component signals. The interferometric signal obtained for the sensing head illustrated above (when no external forces act on it) is presented in Figure 2.

**Figure 3.**
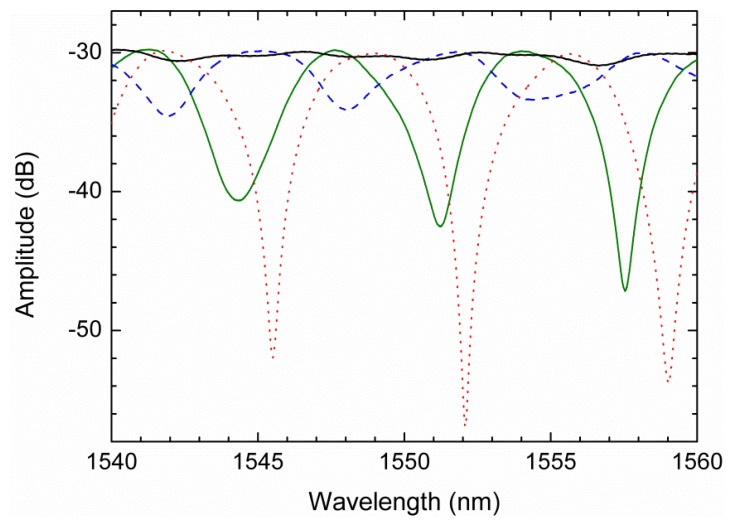
Interferometer spectra obtained for different polarization controller positions.

Since the sensing head is based on a Hi-Bi PCF, it will be sensitive to the angle between the input polarized light and the birefringent axes of the fiber. It is expected from theory that if this angle is 0° or 90° there is no interference signal, however if the angle is 45° both components will have the same optical input and the interference will be maximum. Figure 3 presents the interferometric spectra obtained for different angles between the input light and the birefringent axes. These results were obtained using the setup illustrated in Figure 1.

**Figure 4.**
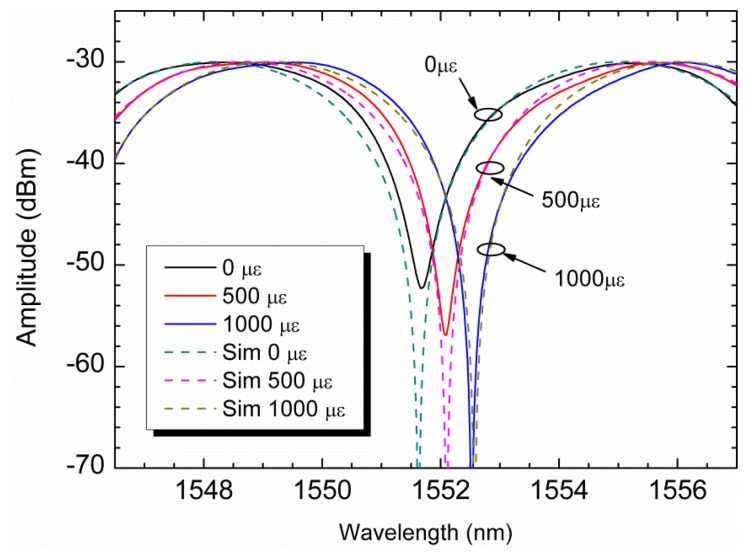
Experimental results (solid line) and theoretical simulations (dash line) of the reflected output signal for three different strain induced variations.

When strain variations are imparted to the PCF sensing head, the interference output signal (presented in Figure 2) will shift in wavelength. Figure 4 displays the experimental and theoretical results obtained for three different strain variations (0 με, 500 με and 1,000 με). As it can be seen in Figure 4, the interferometric spectrum presents a wavelength shift when strain variations are forced into the sensing head, which is quite in agreement with the simulations presented. Based on this characterization, strain measurement can be achieved by monitoring the interference wavelength shift, which presents a proportionality behavior with strain variations.

## 4. Sensor System and Results

**Figure 5.**
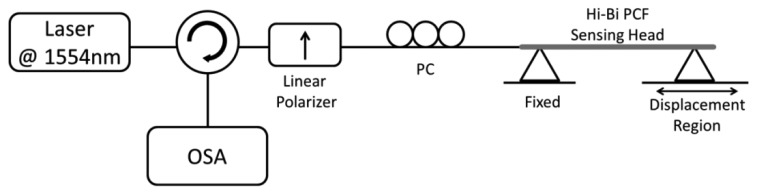
Experimental setup for strain measurement with an interferometric in-reflection Hi-Bi PCF sensing head.

After the characterization of the Hi-Bi PCF sensing head, its response to strain variations was measured through a more accessible, intensity based, setup which is presented in Figure 5. The experimental configuration consisted of a laser working at 1,554 nm (Ando AQ8201-13), a circulator, a linear polarizer, a polarization controller, the Hi-Bi PCF sensing head, and an optical spectrum analyzer (OSA) with a maximum resolution of 10 pm. After passing through the circulator, the laser light is linearly polarized and the polarization angle optimized before reaching the Hi-Bi PCF sensing head. The interferometric reflected signal will make a pass again through the circulator before reaching the OSA.

**Figure 6.**
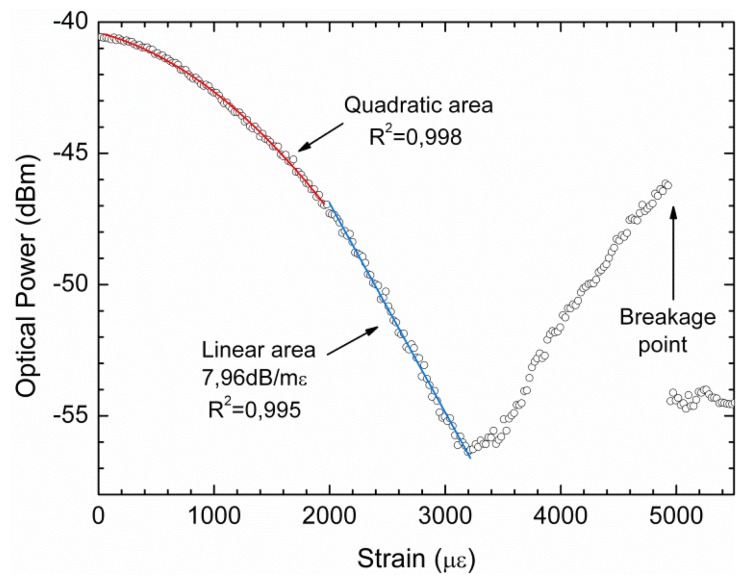
Measured optical power variation with strain.

When strain changes are imparted to the Hi-Bi PCF sensing head, its output signal shifts in wavelength (see Figure 4). Using the cost effective system illustrated in Figure 5, the sensing head interrogation will be made through the laser, and as so in intensity. Since its interrogation is now made with a peak laser the output signal due to strain changes will present power shifts. Since the Hi-Bi PCF sensing head is illuminated by the peak laser, the output signal will be obtained only in the part of the interferometric signal that is in its cone of illumination. As so, if the sensing head signal is in an interferometric minimum the output peak power will be at its minimum value, meanwhile if it is at an interferometric maximum the output peak power will be at its maximum value. This will provide a visual sensation that the laser line is sweeping the interferometric signal, as the output peak power varies between a maximum and a minimum. The observed power shift with strain induced variations is depicted in Figure 6, using a stepper motor with increments of 22.2 με. The Hi-Bi PCF sensing head response showed a quadratic behavior followed by a linear one; this last with a sensitivity to strain variations of 7.96 dB/mε in an operational region of 1,200 με. The rupture point of the sensor head was found to be close to 5,000 με.

The presented sensing head sensitivity to strain induced variations is higher than other developed structures based on this fiber. For instance, when using the Hi-Bi PCF as the sensing element in a fiber loop mirror a sensitivity to strain that varied from 2.7 dB/mε at 1,530 nm to 3.2 dB/mε at 1,545 nm was obtained [9]. This interferometric sensing head shows appropriate response to strain variations, opening the possibility to obtain even better performance with a proper auto-referenced interrogation scheme such as a highly stable in-quadrature dual-wavelength fiber laser [16]. Also, the use of this system in-reflection is an attractive choice as a basic sensing element since it is simple, compact and presents the ability for remote sensing and multiplexing. Even more, reflective sensors enable the possibility for interrogation from a network header using a single fiber, as done in OTDR interrogation systems [17].

## 5. Conclusions

A simple configuration for an interferometric fiber optic strain sensor was presented and experimentally demonstrated. The sensing head is achieved by using a Hi-Bi PCF in-reflection. An experimental characterization of this sensing head was made using an optical backscatter reflectometer, which was in accordance with the presented theoretical simulations. Using a more cost-effective setup, strain variations could be accurately retrieved. The in-reflection sensing head presented a sensitivity of ∼7.96 dB/mε to strain induced variations. Due to the demonstrated strain sensitivity, this interferometric sensing head is a very attractive solution for applications such as strain measurement in hazard environments and health monitoring of complex structures.
